# An entomopathogenic fungus exploits its host humoral antibacterial immunity to minimize bacterial competition in the hemolymph

**DOI:** 10.1186/s40168-023-01538-6

**Published:** 2023-05-20

**Authors:** Jia-Lin Wang, Jing Sun, Ya-Jing Song, Huan-Huan Zheng, Gui-Jie Wang, Wen-Xia Luo, Li Li, Xu-Sheng Liu

**Affiliations:** Hubei Key Laboratory of Genetic Regulation and Integrative Biology, School of Life Sciences, Central China Normal University, Wuhan, 430079 China

**Keywords:** Entomopathogenic fungus, Antimicrobial peptide, Gut microbiota, Competition, *Helicoverpa armigera*

## Abstract

**Background:**

The insect hemolymph (blood-equivalent fluid), composed of a large number of hemocytes (blood cells) and a variety of soluble immune effectors, is hostile for pathogens including fungi. In order to survive in the insect hemocoel (body cavity), the entomopathogenic fungus (EPF) has evolved two classical coping strategies, namely evasion and suppression of the host immune reactions. However, it remains unclear whether EPF has other ways of coping with host immunity.

**Results:**

In this study, we demonstrated that *Metarhizium rileyi* (an EPF) infection by injection of blastospores into the hemocoel enhanced the plasma antibacterial activity of cotton bollworm (*Helicoverpa armigera*), which was partially due to the enhanced expression of antimicrobial peptides (AMPs). The early stage of *M. rileyi* infection induced the translocation of gut bacteria into the hemocoel, where they were subsequently cleared due to the enhanced plasma antibacterial activity. Further, we showed that the enhanced plasma antibacterial activity and AMP expression were attributable to *M. rileyi* but not the invasive gut bacteria (opportunistic bacteria). Elevated ecdysone (major steroid hormone in insects) levels in the hemolymph at 48 h post-*M. rileyi* infection might contribute to the enhanced expression of AMPs. The fungus-elicited AMPs, such as cecropin 3 or lebocin, exhibited potent inhibitory activity against the opportunistic bacteria but not against hyphal bodies. In addition, the opportunistic bacteria competed with hyphal bodies for amino acid nutrients.

**Conclusions:**

*M. rileyi* infection induced the translocation of gut bacteria, and then the fungi activated and exploited its host humoral antibacterial immunity to eliminate opportunistic bacteria, preventing them from competing for nutrients in the hemolymph. Unlike the classical strategies, EPF utilizes to evade or suppress host immunity, our findings reveal a novel strategy of interaction between EPF and host immunity.

Video Abstract

**Supplementary Information:**

The online version contains supplementary material available at 10.1186/s40168-023-01538-6.

## Background

The insects have evolved robust immune systems because of constant exposure to potentially harmful pathogenic organisms. The innate immune system of insects consists of cellular and humoral immunity. Cellular immunity is composed of hemocyte-mediated phagocytosis, nodulation, and encapsulation [1, 2]. Humoral immunity mainly involves the synthesis of antimicrobial peptides (AMPs) in the fat body and their subsequent secretion into the hemolymph, as well as prophenoloxidase activation [1, 2]. Pattern recognition receptors, such as C-type lectins (CTLs), peptidoglycan recognition proteins (PGRPs), and β-1,3-glucan recognition proteins (βGRPs), are responsible for the recognition of conserved surface determinants of the pathogens, thereby triggering innate immune responses [2, 3].

Entomopathogenic fungus (EPF) is a promising biocontrol agent which infects and kills the insect host efficiently [4]. The infection begins with the attachment of EPF conidia to host cuticle, where conidial germ tubes form and subsequently penetrate the cuticle and epithelial tissues. Upon reaching the hemocoel, the germ tubes bud to produce yeast-like hyphal bodies or in vivo blastospores, which replicate extensively [5]. To survive in the insect’s hemocoel, EPF has evolved passive and active strategies to evade and suppress the host’s strong immune reactions, respectively. Passive strategies involve eliminating or masking the components (such as carbohydrate epitopes) of the fungal cell wall to avoid being recognized and immune stimulation [6–8]. Active strategies include the secretion of secondary metabolites (also known as toxins) by EPF to disrupt or suppress the host’s immune reactions [9, 10]. In addition to the above two strategies, whether EPF has other ways of dealing with the host immune responses remains obscure.

Despite the significant advances in the mechanisms of host–pathogen interactions, few studies have examined the effects of host microbes [11]. Gut microbiota is integrated into host biology and physiology and is considered a virtual “organ” [12]. It is essential in modulating host–pathogen interactions. Eliminating gut bacterial flora with antibiotics makes mosquitoes susceptible to the parasite *Plasmodium* infection [13]. The gut bacteria also protect honeybees from invasion by bacterial pathogens [14, 15]. The persistence of gut microbiota affects the host’s susceptibility to pathogens, and conversely, pathogenic infection leads to gut microbiota dysbiosis, whether in mammals or in insects. For instance, the pathogenic *Mycoplasma hyorhinis* infection results in dysbiosis of the gut microbiota and dysfunction of the intestinal barrier in pigs [16]. The peritrophic matrix (PM) is a semipermeable membrane that separates luminal contents from the midgut epithelium in most insects [17]. Infection by a pathogenic bacterium disturbs the tick’s gut microbiota and influences the PM integrity [18]. *Beauveria bassiana* is an EPF that downregulates the expression of AMPs and dual oxidase in the midgut, resulting in dysbiosis of mosquito gut microbiota. A gut bacterium *Serratia marcescens* overgrows in the midgut and then migrates to the hemocoel [19]. These studies suggest that infection by mycoplasma, bacteria, or fungi leads to dysbiosis of host gut microbiota and sometimes translocation of the gut bacteria to hemocoel. Once some gut bacteria migrate into the hemocoel, their roles change from resident symbionts to pathogens and even cause septic death [20]. However, the fate of these gut-derived (opportunistic) bacteria in hemolymph is unknown.

Increasing evidence indicates that various bacteria inhabit the insect’s hemolymph stably or transiently [21–23]. These bacteria require nutrients and may drain the hemolymph’s nutritional resources [23]. In order to grow and complete the lifecycle, the EPF requires nutrients from the hemolymph [24]. Since fungal infection promotes the translocation of bacteria from the gut to hemocoel [19], how does EPF cope with nutritional competition from bacteria? Fan et al. [25] have revealed that the EPF *B. bassiana* oosporein acts as an antibacterial compound, thus limiting bacterial growth after host death and avoiding bacterial competition on insect cadavers. However, the strategies EPF employs to limit bacterial competitors before the host death are still unknown.

We have previously demonstrated that the infection of *Metarhizium rileyi* (also known as *Nomuraea rileyi*) impairs and evades cellular immunity in the lepidopteran insect *Helicoverpa armigera* [26, 27]. In the current study, we found that *M. rileyi* infection stimulated plasma antibacterial activity and enhanced the AMP expression. Further, *M. rileyi* infection induced the translocation of gut bacteria (such as *Acinetobacter johnsonii*) into the hemocoel, which were soon cleared from the hemolymph. The fungus-elicited AMPs contributed to eliminating invasive gut bacteria, which could compete with hyphal bodies for amino acid nutrition in the hemolymph. Understanding the interactions between EPF, host insects, and gut microbiota will be beneficial for developing novel biological pest control strategies.

## Methods

### Insect rearing, antibiotic treatment, and sample preparation

*Helicoverpa armigera* larvae were maintained at 28 ± 1 °C and 70% relative humidity, with a 14-h light/10-h dark photoperiod. Non-axenic larvae were reared with an artificial diet made mainly from wheat germ and soybean powder. Axenic larvae were generated by feeding on the same artificial diet supplemented with gentamicin (15 μg/mL), streptomycin (10 μg/mL), and penicillin (10 units/mL) from the time of the third-instar. The guts were collected from non-axenic and axenic larvae at 1 day post-ecdysis (PE) of sixth-instar larvae. The gut homogenates were plated onto the LB agar plates and incubated at 37 °C overnight to evaluate the efficacy in eliminating cultured bacteria. PCR amplification of the bacterial 16S rRNA gene fragment was performed using universal primers 16S-F and 16S-R (Table S1).

We prepared the samples according to the method described previously [19, 28]. Sixth-instar larvae were surface-sterilized in 75% ethanol and then rinsed three times in sterile PBS. Hemolymph was collected by cutting the abdominal feet of larvae without touching the guts and diluted (1:3) in an anticoagulant. The precipitates (containing hemocytes and microbes) were obtained after centrifugation of hemolymph-anticoagulant mixture at 1000 × *g* for 10 min. The supernatant was further centrifugated at 10,000 × *g* for 10 min to obtain the cell-free plasma. The guts were dissected and rinsed three times in sterile PBS to avoid hemolymph contamination.

### Fungal culture and infection assays

*Metarhizium rileyi* strain NR06 was maintained on SMAY plates [Sabouraud maltose agar medium (1% maltose, 1% peptone, and 1.2% agar) fortified with 1% yeast extract] at 25 °C. After sporulation, the conidia were inoculated into the SMY medium [Sabouraud maltose medium (1% maltose and 1% peptone) fortified with 1% yeast extract] broth and cultured for 60 h with shaking (180 rpm, 25 °C). Subsequently, the blastospores were harvested, resuspended in PBS, and adjusted to 1 × 10^4^/μL using a hemocytometer.

A total of 5 μL blastospore-suspended PBS or sterile PBS (as control) was injected into the hemocoel of each sixth-instar larvae (6–12 h PE). The infection assays were repeated twice, and 30 larvae were used in each treatment of both the axenic and non-axenic group. The mortality was recorded every 6 h, and survival curves were generated.

### Peritrophic matrix (PM) analysis

Since the larvae begin to die at around 72 h post-injection (hpi) of *M. rileyi* blastospores [29], the 12 hpi is considered to be the early stage of infection and used to test the integrity of PM. The midguts were dissected from the larvae at 12 hpi of blastospores or PBS. Three larvae were taken from each treatment and three replicates were performed. We prepared paraffin sections according to the method described previously [30]. After fixing in 4% paraformaldehyde, dehydrating in a series of ethanol grades, and infiltrating with xylene, the midgut samples were embedded in paraffin wax. The samples were sectioned at 5 μm and mounted on gelatin-coated slides. After dewaxing and rehydration, the sections were stained with hematoxylin and eosin (H&E). Finally, the slides were examined and photographed under bright-field illumination of a microscope.

### Transcriptomic sequencing and bioinformatics analysis

To screen the genes involved in PM degradation, we collected the midgut samples from blastospore-injected (MB) or PBS-injected (MP) larvae at the same time point (12 hpi) as PM analysis. These midgut samples were used for transcriptomic analysis. Six cDNA libraries (MB1, 2, 3; MP1, 2, and 3) were constructed and sequenced on the Illumina Novaseq 6000 platform (San Diego, USA) following the standard protocols set by Majorbio Bio-Pharm Technology Co. Ltd. (Shanghai, China). Subsequently, clean reads were mapped to the genome of *H. armigera* (https://www.ncbi.nlm.nih.gov/genome/13316?genome_assembly_id=319039) using TopHat (http://tophat.cbcb.umd.edu/) software. After assembly of the mapped reads, the unigenes were run against a non-redundant database. Differentially expressed genes (DEGs) were determined with a cutoff of |log2 fold change (MB/MP)|≥ 1 and adjusted *p*-value ≤ 0.05, as the criteria used in the previous study [31].

### Microbiota collection, 16S rRNA sequencing, and bioinformatics analysis

The 48 hpi of blastospores represents the middle to late stage of *M. rileyi* infection, when plasma antibacterial activity is significantly enhanced [29]. Therefore, the hemolymph from blastospore-injected (HB) or PBS-injected (HP) larvae at 48 hpi was collected and the precipitates were obtained. Simultaneously, the guts were dissected from the blastospore-injected (GB) or PBS-injected (GP) larvae at 48 hpi. The precipitates or guts were subjected to total metagenomic DNA extraction, followed by amplifying the V3 and V4 hypervariable regions of the 16S rRNA gene pool using the primers 338F and 806R (Table S1). Subsequently, the PCR amplicon libraries (HB1, 2, 3; HP1, 2, 3; GB1, 2, 3; GP1, 2, and 3) were constructed and subjected to sequencing on an Illumina MiSeq platform (San Diego, USA) following the standard protocols set by Majorbio Bio-Pharm Technology Co. Ltd. The resulting raw reads were filtered to obtain clean data. Operational taxonomic units (OTUs) with a 97% similarity cutoff were clustered using UPARSE (http://drive5.com/uparse/). The taxonomy of each OTU representative sequence was analyzed by RDP Classifier (http://rdp.cme.msu.edu/) against the 16S rRNA database (http://www.arb-silva.de) with a confidence threshold of 0.7 [32].

### Isolation, characterization, inoculation of gut bacteria, and infection assays

The gut homogenates were diluted and spread onto the LB agar plates. After incubation at 37 °C for 24–48 h, the morphologically distinct colonies were picked out for genomic DNA extraction using a bacterial DNA kit (Omega Biotech, GA, USA). The DNA fragments were amplified using 16S rRNA gene primers 27F and 1492R (Table S1), followed by sequencing and blasting against the 16S rRNA database. The gut bacterial strains *A. johnsonii* (GenBank no. LN624799), *Stenotrophomonas rhizophila* (GenBank no. NR_121739), and *Enterobacter ludwigii* (GenBank no. NR_042349) were characterized*.* These gut bacteria in the mid-logarithmic phase were harvested and resuspended in PBS (1 × 10^5^ cells/μL). A total of 5 μL of each bacterial suspension was injected into the hemocoel of each sixth-instar larva at 6 − 12 h PE. The infection assays were repeated twice, and 33 larvae were used in each treatment. The mortality was recorded every 12 h, and survival curves were generated.

### Quantification of gut and hemolymph bacteria by quantitative PCR (qPCR)

The genomic DNA of the gut or hemolymph was extracted at 0, 12, and 48 hpi of *M. rileyi* blastospores or PBS using a genomic DNA isolation kit (Omega Biotech). The genomic DNA of the hemolymph was extracted at 12, 24, 36, 48, and 60 hpi of each species of gut-derived bacteria. The quantification of total bacteria, *A. johnsonii*, *S. rhizophila*, or *E. ludwigii* by qPCR was performed on genomic DNA using universal 16S rRNA primers or bacterial-specific 16S rRNA primers (Table S1). The qPCR analysis was conducted using a TransStart Top Green qPCR SuperMix (TransGen Biotech, Beijing, China). The larval housekeeping gene *β-actin* was used as a control, and the quantification was conducted in triplicate.

### Label-free proteomics and bioinformatics analysis

The cell-free plasma was collected from the sixth-instar larvae injected with blastospores (PBL) or PBS (PPB) at 48 hpi. The plasma proteins were extracted using SDT lysis buffer (4% SDS, 0.1 M DTT, 100 mM Tris–HCl, pH 7.6). After quantification of the protein samples using a BCA protein assay kit (Bio-Rad, CA, USA), each sample was digested with trypsin according to the filter-aided sample preparation method [33]. Digested peptides were analyzed by nano-scale liquid chromatography tandem mass spectrometry (LC–MS/MS) using Thermo Scientific EASY column. The MS hit list was curated and annotated for bioinformatics analysis with UniProt (https://www.uniprot.org/) IDs. Differentially expressed proteins (DEPs) were considered significant with a fold cutoff change value ≥ 2 and adjusted *p*-value ≤ 0.05 [34].

### Measurement of ecdysteroid and injection of 20-hydroxyecdysone (20E)

We prepared the sample for ecdysteroid measurement according to a previously described method [35, 36]. Briefly, hemolymph collected from the sixth-instar larvae at 12 and 48 hpi of blastospores or PBS was collected. A total of 50 μL of hemolymph was diluted with chilled methanol, followed by centrifugation at 12,000 × *g* for 10 min. The upper layer of the sample was transferred into a clean tube, dried by evaporation using a SpeedVac, and resuspended in enzyme immunoassay (EIA) buffer. 20E at different concentration gradients was used to generate standard curves. The sample was then assayed by EIA kit (Cayman Chemical, MI, USA) following the manufacturer’s instructions.

We performed 20E treatment according to a previously described method [36]. 20E was dissolved in dimethyl sulfoxide (DMSO) to obtain a 10 mg/mL stock solution, which was further diluted to 40 ng/μL with PBS. To measure AMP expression, each sixth-instar larva at 24 h PE was injected with 20E at 200 ng/5 μL, which was close to the amount of 20E in hemolymph of each larva during the wandering stage [37]. The equivalent amount of DMSO diluted in PBS was injected as control. Three fat bodies from each treatment were pooled together as a replicate (*n* = 3) and the total RNA was extracted at 12, 24, and 48 hpi.

### Reverse transcription quantitative PCR (RT-qPCR)

Total RNA of the midgut and fat bodies was extracted from the sixth-instar larvae injected with blastospores or PBS at 12, 24, and 48 hpi. Also, the total RNA of fat bodies was extracted from the axenic sixth-instar larvae injected with blastospores or PBS at 48 hpi. We also isolated the total RNA of the fat bodies from the larvae injected with each species of gut-derived bacteria or PBS at 24 and 48 hpi, as well as from larvae injected with 20E or DMSO. Primers designed for *chitinase-like protein EN03* (*ChtEN03*, GenBank no. XM 021340696), *gloverin* (GenBank no. KT346373), *cecropin 3* (GenBank no. GU182910), and *lebocin* (GenBank no. KT346375) are listed in Table S1. RT-qPCR was conducted with TransStart Top Green qPCR SuperMix. Quantitative analysis for each gene was conducted in triplicate and normalized against *β-actin* using the 2^−ΔCT^ calculation method.

### Analysis of the antibacterial activity of plasma and AMPs

Mid-logarithmic bacteria, such as *Escherichia coli* (*E. coli*), *Staphylococcus aureus* (*S. aureus*), and three gut-derived bacteria, were pelleted and resuspended in PBS at a concentration of 1 × 10^5^ cells/μL. To examine whether and at which stage *M. rileyi* infection enhanced the plasma antibacterial activity, hemolymph from non-axenic larvae was collected at 12 and 48 hpi of blastospores or PBS. To compare the plasma antibacterial activity, hemolymph from non-axenic and axenic larvae at 48 hpi of blastospores or PBS was harvested. To determine the antibacterial activity of the plasma, 90 μL of plasma was mixed with 10 μL of the bacterial suspension. After incubation at 25 °C for 1 h, the plasma-bacterial mixture was plated onto the LB agar plates and cultured at 37 °C for 24–48 h. The number of colonies on each plate was recorded.

A C-terminal amidated peptide (RWKVFKKIEKVGRNIRDGVIKAGPAIEVLGQAKAI) corresponding to the 27–61 amino acids of cecropin 3, as well as a peptide (SLHLPGYDFPLPPFNPRPRYPWDEKP) corresponding to the 125–153 amino acids of lebocin, was synthesized and purified by high-performance liquid chromatography, GenScript Co. Ltd (Nanjing, China). The cecropin 3 or lebocin peptides were dissolved in sterile water to desired concentrations. To measure their antibacterial activity, 90 μL of cecropin 3 (25 μM) or lebocin (100 μM) was mixed with 10 μL of each bacterial suspension, followed by incubation at 25 °C for 1 h. Next, the mixture was plated onto the LB agar plates and cultured at 37 °C for 24–48 h. The same concentration of BSA or sterile water was used as a control. The number of colonies on each plate was recorded, and the measurement was conducted three times.

### Analysis of the antifungal activity of AMPs

The hemolymph was collected from the larvae at 48 h post-blastospore injection and diluted (1:3) in an anticoagulant. After centrifugation of the mixture at 1000* g* for 10 min, a pellet (containing hemocytes and hyphal bodies) was obtained. Sterile water was added, and hemolysis occurred, followed by centrifugation at 1000* g* for 10 min. After repeating the above steps three times, hyphal bodies were identified by their morphological characteristics under a microscope and collected for further assays.

We adjusted the concentration of cecropin 3 and lebocin to 100 and 200 μM, respectively, which was higher than that used in antibacterial assays. To test the antifungal activity, 90 μL of cecropin 3 or lebocin was mixed with 10 μL of hyphal body suspension, followed by incubation at 25 °C for 1 h. Next, the mixture was dropped onto SMAY plates and cultured for 6 days. The same concentration of BSA or sterile water was used as a negative control. Benomyl (10 mg/mL) was used as a positive control. The diameter of the fungal colony was measured, and the tests were repeated three times.

### Bacterial-fungal co-culturing assays

We performed the co-culturing assays according to a previously described method [25]. Briefly, the *M. rileyi* conidia (final concentration, 4 × 10^4^ conidia/μL) were inoculated into 5 mL of 0.5 × SMY medium with or without *A. johnsonii* (final concentration, 0.5 × 10^4^ cfu/μL). After incubation at 25 °C for 84 h, the samples were examined and photographed under bright-field illumination of a microscope.

### Hyphal body and total hemocyte counts

Hemolymph was collected from sixth-instar larvae at 12, 24, 36, 48, and 60 hpi of blastospores or PBS. A total of 5 μL of *A. johnsonii*-suspended PBS (concentration, 1 × 10^3^, 1 × 10^4^, or 1 × 10^5^ cfu/μL) or PBS (control) was injected into the hemocoel of sixth-instar larvae pretreated with blastospores. The hemolymph was collected at 12 and 24 hpi of *A. johnsonii* or PBS, corresponding to the 60 hpi of blastospores. Hyphal bodies were checked, photographed, and counted under the bright-field illumination of a microscope. The number of total hemocytes was recorded with the help of a hemocytometer.

### Measurement of the total free amino acid concentration

The plasma was collected from the sixth-instar larvae at 12, 48, and 60 hpi of blastospores or PBS, as well as from sixth-instar larvae at 60 hpi of each species of gut-derived bacteria or PBS. These plasma samples were transferred to clean tubes, followed by addition of extraction buffer. After boiling for 15 min, the mixture was cooled and centrifuged at 10,000* g* for 10 min. Subsequently, the upper layer of the sample was assayed by ninhydrin colorimetry (Abbkine, Wuhan, China) following the manufacturer’s instructions. The total free amino acid concentration in each sample was determined at 570 nm with ninhydrin as the developer. The amino acid measurements were conducted in triplicate.

### Statistical analysis

Statistical analysis was performed using GraphPad Prism software (GraphPad Inc., La Jolla, CA, USA). Student’s *t* test was used for two-group comparisons unless otherwise stated. Data comprising more than two groups were analyzed using one-way analysis of variance (ANOVA) coupled with Tukey’s multiple comparison test. Survival curves were analyzed using the log-rank (Mantel-Cox) test. The difference of bacterial composition between the two groups was analyzed by the Mann–Whitney *U* test. A value of *p* < 0.05 was considered significantly different. The statistical details were indicated in the corresponding figure legends.

## Results

### Enhanced plasma antibacterial activity at 48 h post-*M. rileyi* infection

**Fig. 1:**
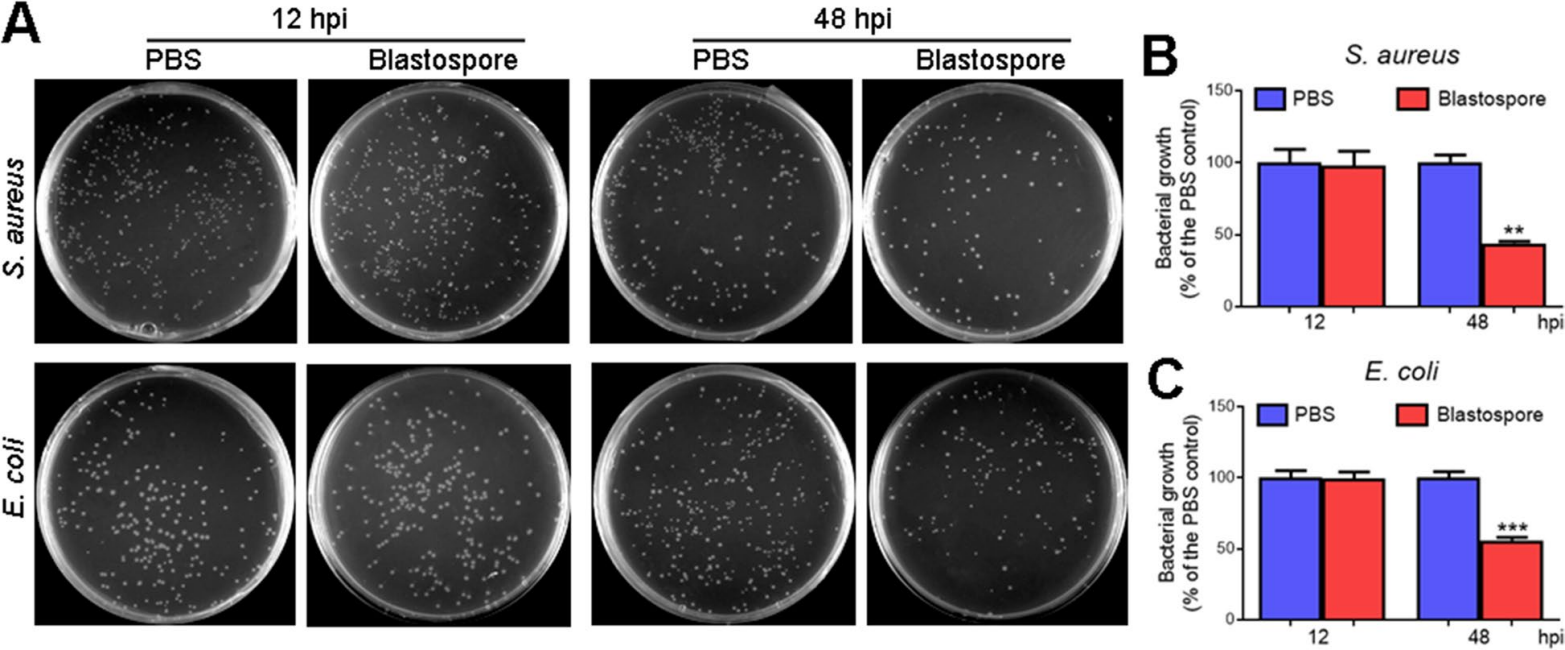
*M. rileyi* infection enhances the plasma antibacterial activity at 48 hpi. **A** The representative LB agar plates showed the colonies of *S. aureus* or *E. coli*. Either *S. aureus* or *E. coli* were incubated with the plasma from the larvae at 12 and 48 h post-injection (hpi) of *M. rileyi* blastospores or PBS (control). The mixture was diluted and plated onto the LB agar plates. **B**, **C** The number of *S. aureus* (**B**) and *E. coli* (**C**) colonies at 12 and 48 hpi was determined. Bacterial growth was exhibited as the ratio of viable colonies against the PBS control group. The data represent the mean ± standard error (SE) for three biological replicates. The statistical differences were analyzed using Student’s *t* test (**p < 0.01 and ***p < 0.001)

To investigate whether *M. rileyi* infection influences the plasma antibacterial activities, the plasma samples collected from *H. armigera* larvae at 12 and 48 h post-injection (hpi) of blastospores or PBS were applied for evaluation. As shown in Fig. 1A–C, the plasma antibacterial activities against *S. aureus* or *E. coli* remained unchanged at 12 hpi; however, the plasma antibacterial activity of blastospore-injected larvae was significantly higher than that of PBS-injected larvae at 48 hpi.

### Enhanced expression of AMPs at 48 h post-*M. rileyi* infection

**Fig. 2:**
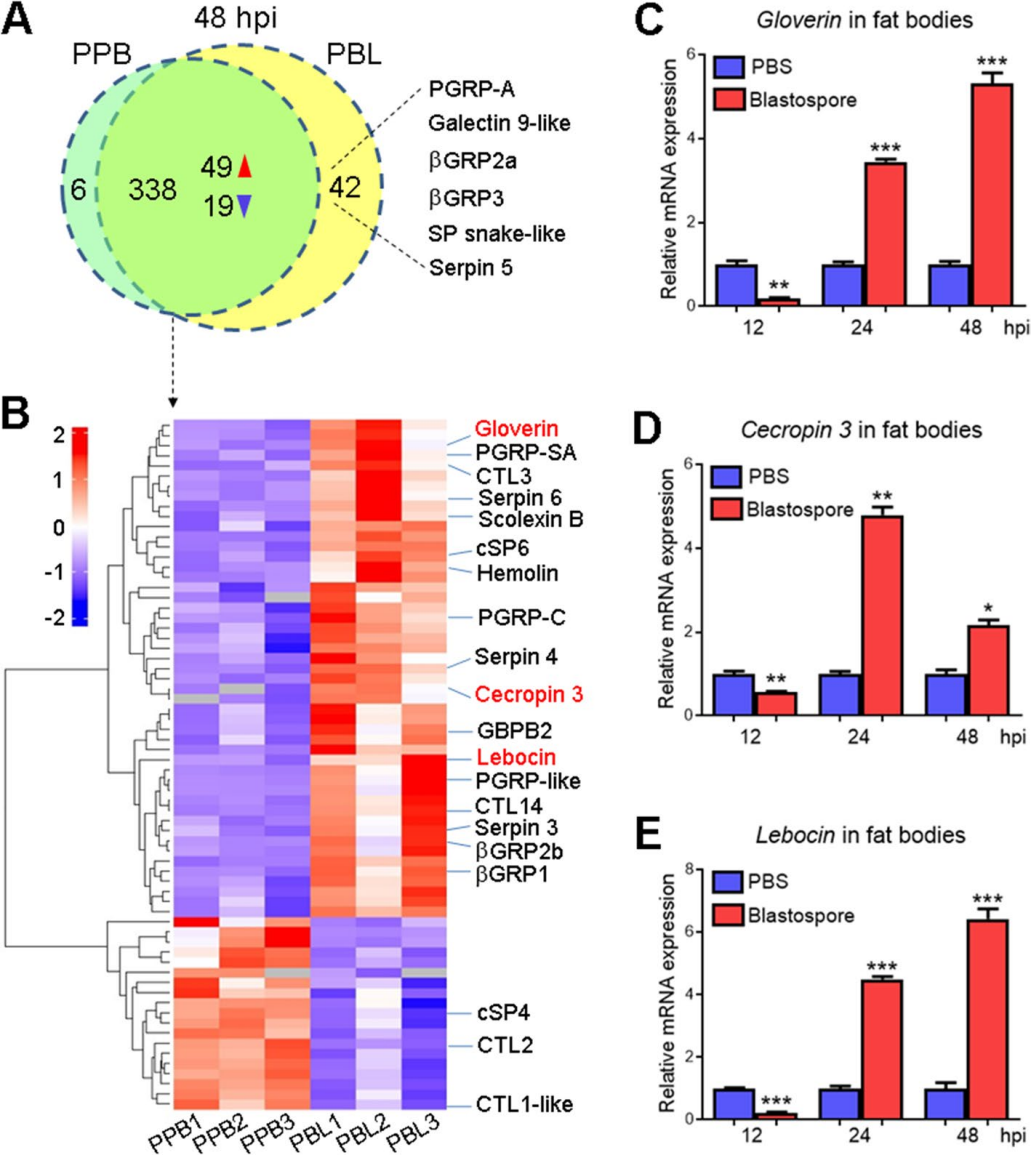
*M. rileyi* infection activates the expression of three AMP genes at 48 hpi. **A** Unique and common proteins between the plasma collected from PBS (PPB)-injected and blastospore (PBL)-injected groups identified by LC–MS/MS analysis. The immune-related proteins unique to PBL are shown. The red and blue arrowheads represent the upregulated and downregulated expression, respectively. **B** Hierarchical clustering analysis of differentially expressed proteins (DEPs) shared in both PPB and PBL groups. All immune-related DEPs are indicated and AMPs are in red. **C-E** The RT-qPCR analysis showed that the expression of *gloverin* (**C**), *cecropin 3* (**D**), and *lebocin* (**E**) was induced in the fat bodies from the larvae at 24 and 48 hpi of *M. rileyi* blastospores. The statistical differences were analyzed using Student’s *t* test (*p < 0.05, **p < 0.01, and ***p < 0.001)

Since the plasma antibacterial activity was significantly enhanced at 48 h post-*M. rileyi* infection, the changes in protein abundance of cell-free plasma were evaluated. Plasma collected from PBS (PPB) or blastospore (PBL)-injected larvae at 48 hpi was subjected to label-free quantitative proteomic analysis. After assembly of peptides into protein sequences, a total of 386 proteins were identified, including 338 shared between PPB and PBL groups, 6 unique to PPB, and 42 exclusively detected in PBL. Among the 42 unique proteins in PBL, several immune-related proteins were identified, such as PGRP-A, galectin 9-like, βGRP2a, βGRP3, serine protease (SP) snake-like, and serpin 5 (Fig. 2A; Table S2). Among the 338 common proteins, 68 DEPs were screened, including 49 upregulated and 19 downregulated in PBL vs. PPB (Fig. 2A, B; Table S3). Among 68 DEPs, 20 immune-related DEPs were identified, most exhibiting evident upregulation (Fig. 2B). PRRs, such as PGRP-SA, PGRP-C, PGRP-like, CTL3, CTL14, βGRP1, βGRP2b, and hemolin, increased their protein abundance. However, CTL2 and CTL1-like decreased their abundance in PBL. The abundance of scolexin B, cSP6, serpin 3, 4, and 6 were increased, whereas cSP4 decreased its abundance in PBL vs. PPB.

The protein abundance of three AMPs (gloverin, cecropin 3, and lebocin) was increased in PBL (Fig. 2B; Table S3), which was consistent with the enhanced plasma antibacterial activity at 48 hpi. The RT-qPCR analysis further indicated that the expression of *gloverin*, *cecropin 3*, and *lebocin* were slightly inhibited at 12 h but were significantly induced at 24 and 48 h post-blastospore injection in the fat bodies (Fig. 2C–E). The induced AMPs in the fat bodies might be secreted into the hemolymph and might enhance the plasma antibacterial activity at 48 hpi.

### Translocation of gut bacteria into the hemocoel at 12 h post-*M. rileyi* infection

**Fig. 3:**
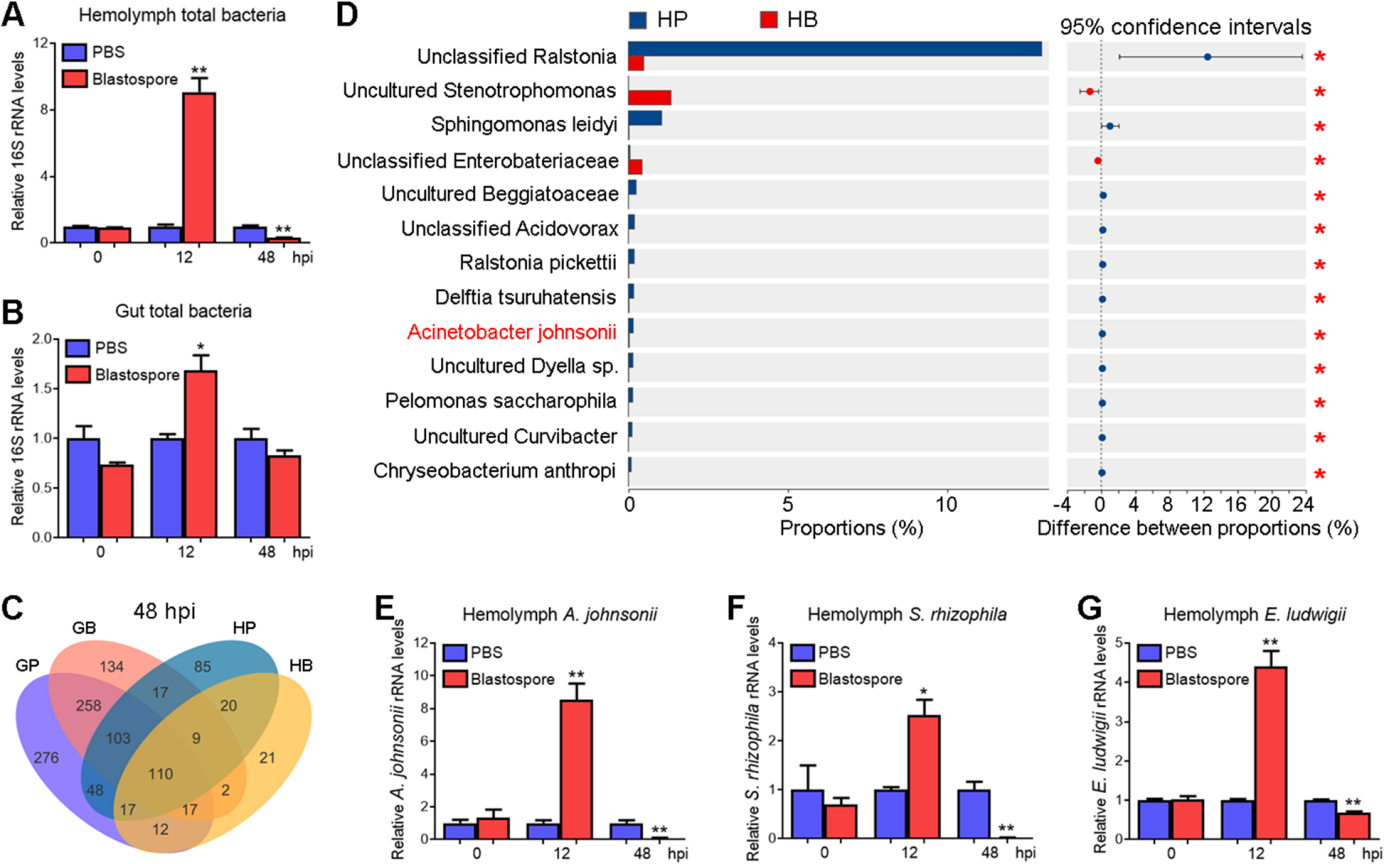
The load of gut-derived bacteria in the hemolymph increases at 12 h and decreases at 48 h post-*M. rileyi* infection. **A**, **B** The quantification of total bacterial load in the hemolymph (**A**) and gut (**B**) of *H. armigera* larvae at 0, 12, and 48 hpi of *M. rileyi* blastospores or PBS. The quantitative PCR analysis was performed against the bacterial genomic DNA using universal primers of the bacterial 16S rRNA gene. **C** Analysis of the hemolymph and gut bacterial operational taxonomic units (OTUs) from the larvae at 48 hpi. HB and HP indicate hemolymph from blastospore-injected and PBS-injected larvae, respectively. GB and GP represent guts from blastospore-injected and PBS-injected larvae, respectively. **D** Analysis of bacterial (species level) abundance in the hemolymph from larvae at 48 hpi. The most abundant 13 species are shown. Gut-derived *A. johnsonii* is in red. **E-G** Quantification of hemolymph *A. johnsonii* (**E**), *S. rhizophila* (**F**), and *E. ludwigii* (**G**) load from the larvae at 0, 12, and 48 hpi of *M. rileyi* blastospores or PBS. Quantitative PCR analysis was performed using specific primers of bacterial 16S rRNA gene and bacterial genomic DNA in the hemolymph as templates. The statistical differences were analyzed using the Mann–Whitney *U* test (*p < 0.05) or Student’s *t* test (*p < 0.05 and **p < 0.01)

Previous studies revealed that fungal infection led to dysbiosis of gut microbiota and caused the translocation of gut bacteria into the hemocoel [19, 38]. To test whether *M. rileyi* infection induces the translocation of gut bacteria into the hemocoel, the hemolymph and gut samples were collected from the larvae at an early stage of blastospore injection. The qPCR analysis indicated that the total bacterial load of the hemolymph or gut was significantly higher in blastospore-injected larvae than in PBS-injected larvae at 12 hpi (Fig. 3A, B). These suggested that *M. rileyi* infection affected the homeostasis of the larval gut microbiota, which may partially result in an increased abundance of hemolymph bacteria at 12 hpi.

As PM lines the midgut lumen and prevents luminal contents (such as gut microbes) from directly contacting with gut epithelial cells [17, 39], we next examined PM structural integrity by H&E staining. A fragmented and inconsecutive PM was visualized in the larvae at 12 h post-blastospore injection, while an intact and consecutive PM existed in the controls (Figure S1A). Transcriptomic analysis indicated that 385 DEGs were upregulated while 177 DEGs were downregulated in the midgut at 12 hpi (Figure S1B; Table S4). Among the total 562 DEGs, a *chitinase-like protein EN03* (*ChtEN03*, LOC110380650) increased its transcript abundance in the midgut after blastospore injection (Table S4). The RT-qPCR analysis further demonstrated that *ChtEN03* was upregulated in the midgut from larvae at 12, 24, and 48 h post-blastospore injection (Figure S1C). Given that chitinase is involved in PM degeneration [40], we hypothesized that ChtEN03 might contribute to the impaired PM in blastospore-injected larvae. Therefore, we concluded that the increased total bacterial load in hemolymph upon *M. rileyi* infection at 12 hpi was partly due to the translocation of gut bacteria.

### Decrease in the gut-derived bacterial load and diversity of the hemolymph at 48 h post-*M. rileyi* infection

Since *M. rileyi* infection caused the translocation of gut bacteria into the hemocoel, the variation in the bacterial load of the hemolymph was analyzed. The qPCR analysis indicated that the total bacterial load of the hemolymph was significantly lower in blastospore-injected larvae than in PBS-injected larvae at 48 hpi (Fig. 3A). This was consistent with the enhanced plasma antibacterial activity and AMP expression at 48 hpi. No significant difference was observed in the gut total bacterial load between blastospore-injected and PBS-injected larvae at 48 hpi (Fig. 3B).

To compare the composition and diversity of microbiota in the gut or hemolymph between blastospore-injected and PBS-injected larvae, the samples at 48 hpi were subjected to deep-sequencing analysis. The number of OTUs in the gut from blastospore-injected larvae was much less than that from PBS-injected larvae, with 162 unique OTUs in the former and 353 unique OTUs in the latter. A similar trend in the OTU number was observed in the hemolymph between blastospore-injected and PBS-injected larvae, with 52 unique OTUs in the former and 253 unique OTUs in the latter (Fig. 3C). These suggested that the gut or hemolymph consists of less bacterial diversity after *M. rileyi* infection. Most bacteria, such as *A. johnsonii*, decreased their abundance in the hemolymph of blastospore-injected larvae (Fig. 3D; Table S5). The 1632 total OTUs were annotated into 40 phyla, 111 classes, 249 orders, 412 families, 729 genera, and 1129 species (Table S5).

There were 278 OTUs shared between the gut and hemolymph from PBS-injected larvae, as well as 138 OTUs shared from blastospore-injected larvae (Fig. 3C). These further suggested that many hemolymph bacteria might come from the gut. To investigate the variation of gut-derived bacteria in hemolymph upon *M. rileyi* infection, we first tried to isolate and characterize the bacteria. By plating gut homogenates onto the LB agar plates, picking single colonies, and sequencing, we eventually isolated and characterized three species of bacteria, namely *A. johnsonii*, *S. rhizophila*, and *E. ludwigii*. The qPCR analysis revealed that the load of hemolymph *A. johnsonii*, *S. rhizophila*, and *E. ludwigii* was upregulated at 12 hpi and downregulated at 48 hpi (Fig. 3E–G), consistent with the variation of hemolymph total bacterial load (Fig. 3A). These results further supported that gut bacteria translocated into hemocoel at 12 hpi and were eliminated mainly at 48 hpi. This elimination might be attributed to the enhanced plasma antibacterial activity and AMP expression at 48 hpi.

### No significant effect of gut microbiota on *M. rileyi* pathogenesis in *H. armigera* larvae

**Fig. 4:**
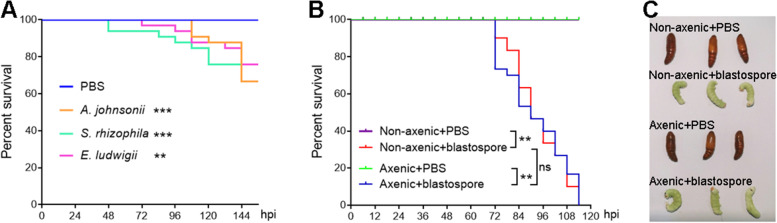
No significant difference in the survival rate was observed between the axenic and non-axenic larvae after *M. rileyi* infection. **A** Survival curves of *H. armigera* larvae injected with *A. johnsonii*, *S. rhizophila*, *E. ludwigii*, or PBS (as control). **B** Survival curves of the axenic and non-axenic larvae injected with *M. rileyi* blastospores or PBS (as control). **C** Representative phenotypes of the axenic and non-axenic larvae injected with blastospores or PBS. *H. armigera* was photographed at 156 hpi. Asterisks represent significant differences (**p < 0.01 and ***p < 0.001) for pairwise comparisons by log-rank test. ns, no significant difference

By injecting *A. johnsonii*, *S. rhizophila*, or *E. ludwigii* into the hemocoel of the larvae, we observed that either of the bacteria significantly decreased the survival rate of *H. armigera* (Fig. 4A). This suggested that translocation of gut bacteria into the hemocoel would accelerate *H. armigera* larval mortality. However, given that those gut-derived bacteria in hemolymph were largely diminished at 48 h post-*M. rileyi* infection, we inferred that the influence of invasive gut bacteria might be eliminated. To test whether gut bacteria affect the insecticidal efficiency of *M. rileyi*, we obtained axenic *H. armigera* larvae via treatment with oral antibiotics. We then confirmed the efficiency of gut bacterial elimination by spreading gut homogenates onto the LB agar plates (Figure S2A) or by conducting PCR assays using universal primers for the bacterial 16S rRNA gene (Figure S2B). By injecting *M. rileyi* blastospores or PBS into the hemocoel of *H. armigera* larvae, we showed that all the larvae died at 72–114 h post-blastospore injection. In contrast, none of the larvae died after PBS injection. As expected, there was no significant difference in the survival rate between the axenic and non-axenic larvae injected with blastospores (Fig. 4B). We further confirmed that blastospore-injected larvae produced conidia on the surface of their mummified cadavers. In contrast, PBS-injected larvae pupated in axenic or non-axenic group (Fig. 4C). These results indicated that gut microbiota did not influence *M. rileyi* pathogenesis in *H. armigera* larvae, further supporting that invasive gut bacteria were removed efficiently from the hemolymph at a later stage of *M. rileyi* infection.

### Enhanced antibacterial activity of plasma and AMP expression at 48 hpi may be attributable to *M. rileyi* but not invasive gut bacteria

**Fig. 5:**
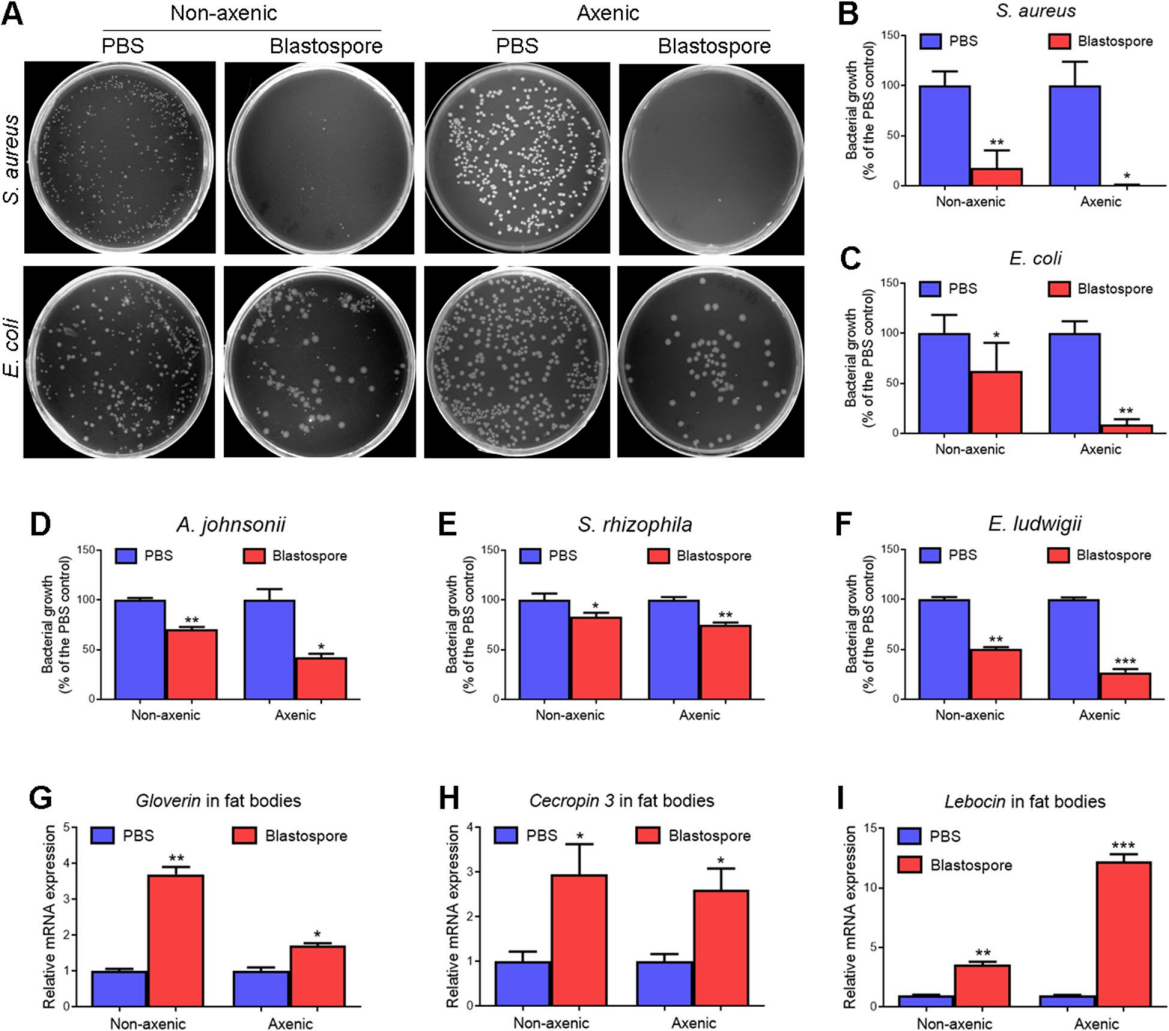
Enhanced plasma antibacterial activity and AMP expression upon *M. rileyi* infection from non-axenic or axenic larvae. **A** After each treatment, the representative LB agar plates showed the *S. aureus* or *E. coli* colonies. Either *S. aureus* or *E. coli* were incubated with the plasma from non-axenic and axenic larvae at 48 hpi of blastospores or PBS. The mixture was diluted and plated onto the LB agar plates. **B-F** The number of *S. aureus* (**B**), *E. coli* (**C**), *A. johnsonii* (**D**), *S. rhizophila* (**E**), and *E. ludwigii* (**F**) colonies after each treatment was determined. Bacterial growth was exhibited as the ratio of viable colonies against the PBS control group. The data represent mean ± standard error (SE) for three biological replicates. **G**-**I** The RT-qPCR analysis showed that the expression of *gloverin* (**G**), *cecropin 3* (**H**), and *lebocin* (**I**) was induced in the fat bodies either from the non-axenic or axenic larvae at 48 hpi. The statistical differences were analyzed using Student’s *t* test (*p < 0.05, **p < 0.01, and ***p < 0.001)

Since *M. rileyi* infection triggers the translocation of gut bacteria into the hemocoel, we wondered whether it was *M. rileyi* or invasive gut bacteria that stimulated the expression of AMPs and enhanced the antibacterial activity. To test this, we first obtained the axenic larvae and confirmed the efficiency of gut bacterial elimination. The plasma antibacterial activities against *S. aureus*, *E. coli*, or three gut-derived bacteria (*A. johnsonii*, *S. rhizophila*, and *E. ludwigii*) were significantly higher in blastospore-injected larvae than that in PBS-injected larvae, either in the non-axenic or axenic group (Fig. 5A–F). Given that plasma antibacterial activity was enhanced upon blastospore injection in the axenic group, we hypothesized that the enhanced antibacterial activity was partially due to the fungus.

Further, the RT-qPCR analysis indicated that the expression of *gloverin*, *cecropin 3*, and *lebocin* was significantly induced in the fat bodies from either non-axenic or axenic larvae at 48 h post-blastospore injection (Fig. 5G–I). Therefore, the enhanced expression of AMP genes in the fat bodies was partially due to the fungus. In this case, we wanted to understand the involvement of invasive gut bacteria. As shown in Figure S3A–C, the gut bacterial challenges suppressed the expression of *gloverin*, *cecropin 3*, and *lebocin* at 24 and 48 hpi in whole. Given that *M. rileyi* infection activates the expression of these AMPs at 24 and 48 hpi (Fig. 2C–E), we believed it was the fungus rather than invasive gut bacteria that activated the expression of AMPs at 48 hpi.

### *M. rileyi* infection activates the expression of AMPs by elevating hemolymph ecdysone levels

**Fig. 6:**
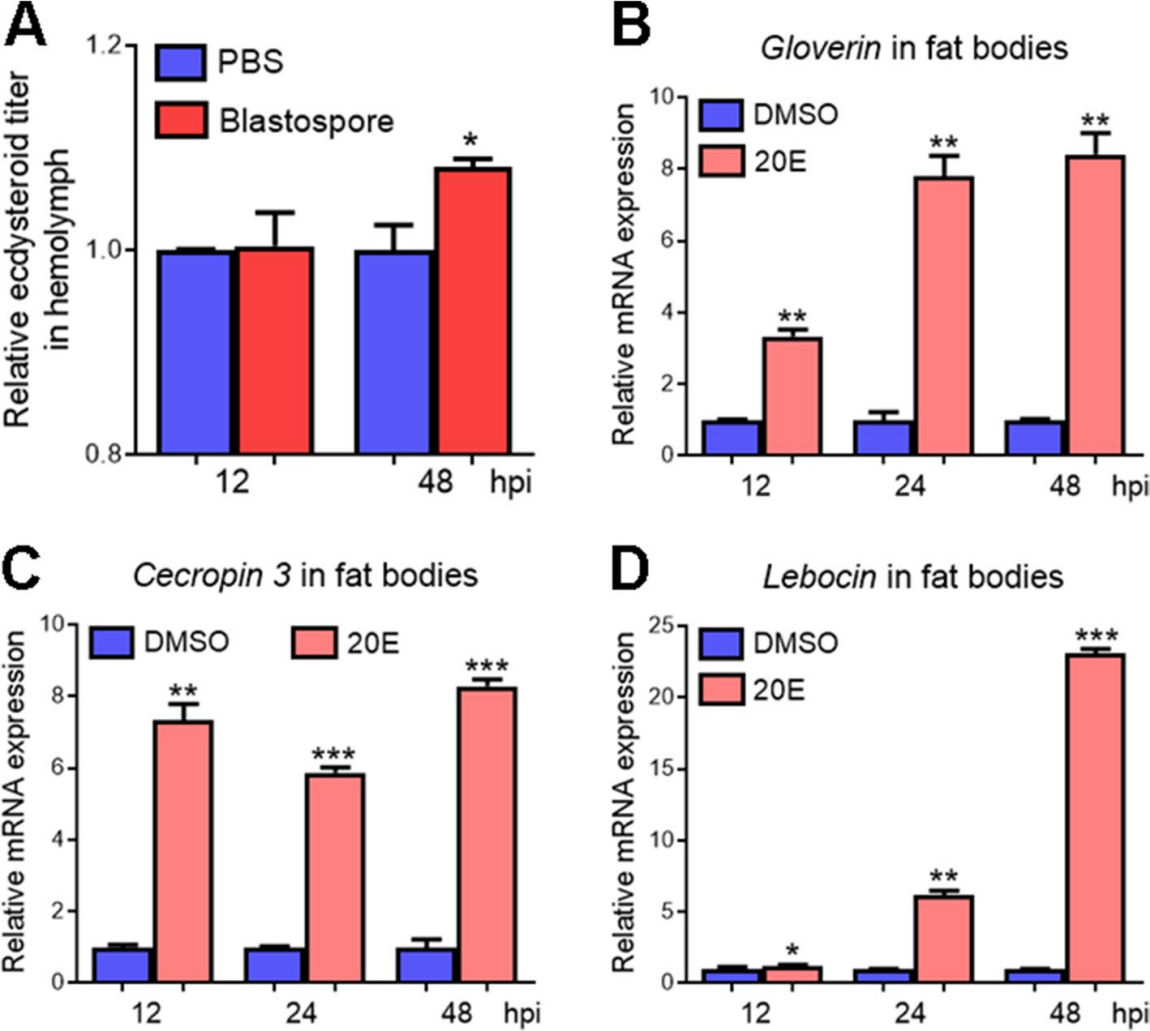
*M. rileyi* infection elevates the hemolymph ecdysone titers, which may activate the expression of AMPs. **A** Measurement of the relative ecdysteroid titers in the hemolymph from larvae at 12 and 48 hpi of blastospores or PBS. **B-D** RT-qPCR analysis of *gloverin* (**B**), *cecropin 3* (**C**), and *lebocin* (**D**) transcripts in the fat bodies of larvae at 12, 24, and 48 hpi of 20E or DMSO. The statistical differences were analyzed using Student’s *t* test (*p < 0.05, **p < 0.01, and ***p < 0.001)

Previous studies have demonstrated that fungal infections alter the insect ecdysone levels [41], and ecdysone functions critically in modulating the expression of immune-related genes and immune reactions [42, 43]. To investigate whether *M. rileyi* infection alters the ecdysone levels, the hemolymph collected from larvae at 12 and 48 hpi of blastospores or PBS was used for the measurement. As shown in Fig. 6A, the hemolymph ecdysteroid titers remained unchanged at 12 hpi; however, they were significantly higher in blastospore-injected larvae than those of the control group at 48 hpi. In addition, the abundance of *gloverin*, *cecropin 3*, and *lebocin* transcripts in the fat bodies were significantly upregulated at 12, 24, and 48 h post-20E injection (Fig. 6B–D). These results indicated that *M. rileyi* infection might stimulate the expression of AMPs by increasing the ecdysone titers in the hemolymph.

### *M. rileyi*-elicited AMPs inhibit gut-derived bacterial growth but cannot suppress the proliferation of hyphal bodies

**Fig. 7:**
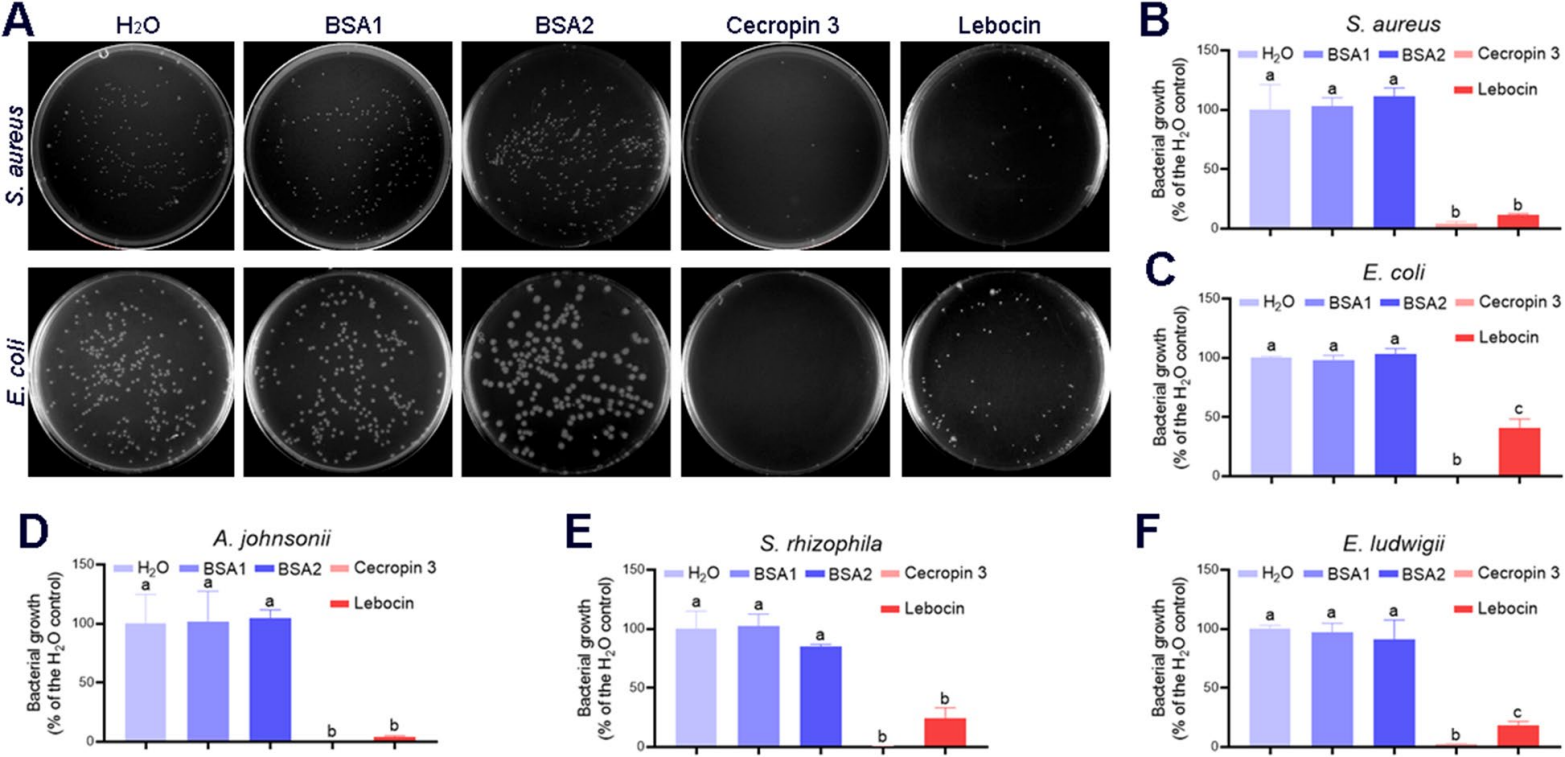
Antibacterial activity of cecropin 3 or lebocin. **A** After each treatment, the representative LB agar plates showed the *S. aureus* or *E. coli* colonies. Either *S. aureus* or *E. coli* were incubated with H_2_O, BSA1 (25 μM), BSA2 (100 μM), cecropin 3 (25 μM), or lebocin (100 μM) at 25 °C with agitation for 1 h. The mixture was diluted and plated onto the LB agar plates. **B-F** The number of *S. aureus* (**B**), *E. coli* (**C**), *A. johnsonii* (**D**), *S. rhizophila* (**E**), and *E. ludwigii* (**F**) colonies after each treatment was determined. Bacterial growth was exhibited as the ratio of viable colonies against the H_2_O control group. BSA was incubated as a control. The data were analyzed using one-way ANOVA followed by Tukey’s multiple comparison test. The different characters above the bars represent significant differences

The enhanced AMP expression and decreased abundance of gut-derived bacteria in the hemolymph at 48 hpi suggested that AMPs contribute to suppressing these bacteria. To test this, we commercially synthesized the active fragments of cecropin 3 and lebocin according to previous reports [44, 45]. We determined the antibacterial activity by incubating these peptides with various bacteria and then plating them onto the LB agar plates. As shown in Fig. 7A–F, incubation with cecropin 3 or lebocin significantly suppressed bacterial growth, including *S. aureus*, *E. coli*, *A. johnsonii*, *S. rhizophila*, and *E. ludwigii*. These results suggested that *M. rileyi*-elicited AMPs contributed to the clearance of invasive gut bacteria in the hemolymph at 48 hpi.

**Fig. 8:**
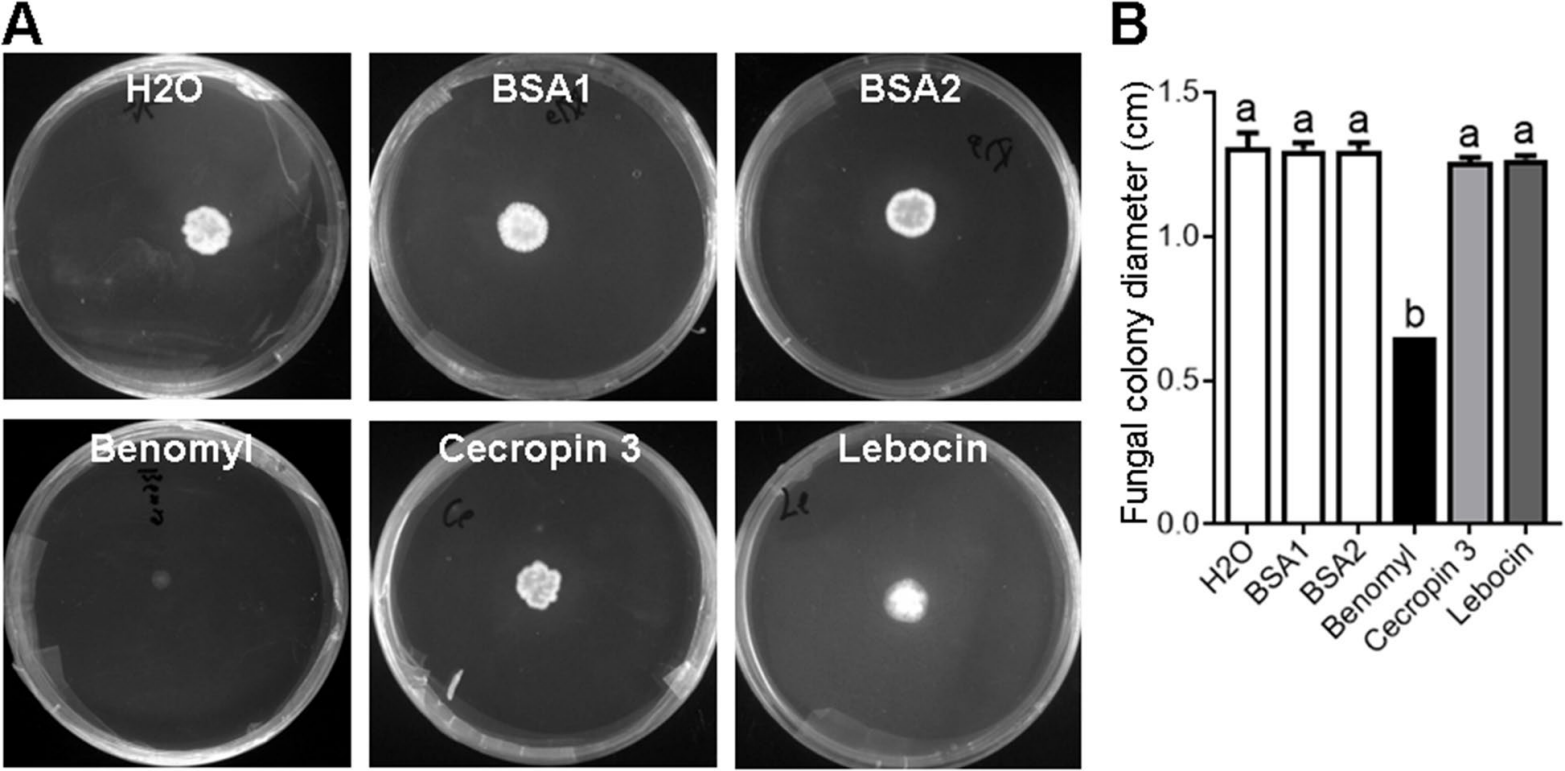
No inhibition activity of cecropin 3 or lebocin against the *M. rileyi* hyphal bodies. **A** The representative SMAY plates showed the fungal colonies after each treatment. The hyphal bodies were isolated from the hemolymph at 48 hpi and then incubated with H_2_O, BSA1 (100 μM), BSA2 (200 μM), benomyl (10 mg/mL), cecropin 3 (100 μM), or lebocin (200 μM). After incubation at 25 °C for 1 h, the mixture was dropped onto the SMAY plates and cultured for 6 days. **B** The diameter of fungal colonies after each treatment was determined. The data were analyzed using one-way ANOVA followed by Tukey’s multiple comparison test. The different characters above the bars represent significant differences

Next, we investigated whether these AMPs could influence the proliferation of *M. rileyi* hyphal bodies. By separating hyphal bodies from the hemolymph of larvae at 48 hpi of blastospores and incubating them with each AMP, we dropped the mixture onto SMAY plates and subsequently measured the diameter of fungal colony. As a positive control, benomyl incubation significantly suppressed the growth of fungal colonies. However, as with negative controls (H_2_O or BSA), incubation with cecropin 3 or lebocin showed no inhibitive activities against the growth of fungal colonies (Fig. 8A, B).

### Gut-derived bacteria compete with hyphal bodies for amino acids in the host hemolymph

**Fig. 9:**
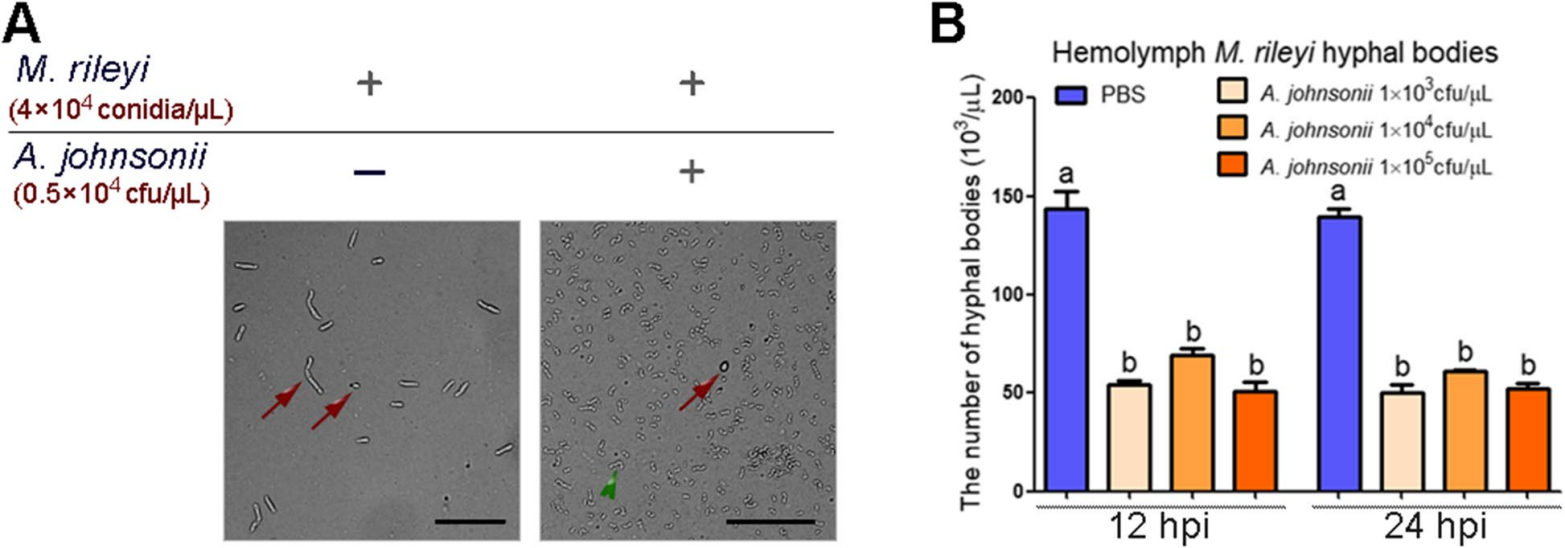
The competition between *M. rileyi* and the gut-derived bacteria. **A** In vitro proliferation of *M. rileyi* and *A. johnsonii* after co-inoculation into the 0.5 × SMY medium at the indicated concentrations. The red arrows indicate *M. rileyi* blastospores, while the green arrowhead points to *A. johnsonii*. Scale bar = 50 μm. **B** The number of hyphal bodies in the hemolymph from the larvae at 12 and 24 hpi of *A. johnsonii* or PBS (control) was determined. The larvae pretreated with blastospores were injected with *A. johnsonii* at a concentration of 1 × 10^3^, 1 × 10^4^, or 1 × 10^5^ cfu/μL. The statistical differences were analyzed using one-way ANOVA followed by Tukey's multiple comparison test. The different characters above the bars represent significant differences

Since *M. rileyi*-elicited AMPs inhibit gut-derived bacterial growth, we wondered its biological significance. To investigate whether bacteria compete with fungi, *A. johnsonii* was co-inoculated with *M. rileyi* conidia in the SMY medium. Co-inoculation of *M. rileyi* with *A. johnsonii* led to bacterial proliferation that overwhelmed the fungi, even though the bacteria started at a lower concentration than the fungi (Fig. 9A). To evaluate the influence of bacteria on the proliferation of fungi in hemocoel, we injected *A. johnsonii*-suspended PBS or PBS (control) into the hemocoel of the sixth-instar larvae pretreated with blastospores. Then, we checked the load of hyphal bodies at 12 and 24 hpi of the bacteria. Interestingly, injection of *A. johnsonii* suppressed the proliferation of hyphal bodies, with a lower load of hyphal bodies in the hemolymph compared to the control group (Fig. 9B). Our in vitro and in vivo experiments confirmed the competition between *M. rileyi* and the invasive gut bacteria. Since there was no significant difference in the inhibitory effect of different doses of bacteria on fungal proliferation, we speculated that the inhibitory effect may have been saturated.

**Fig. 10:**
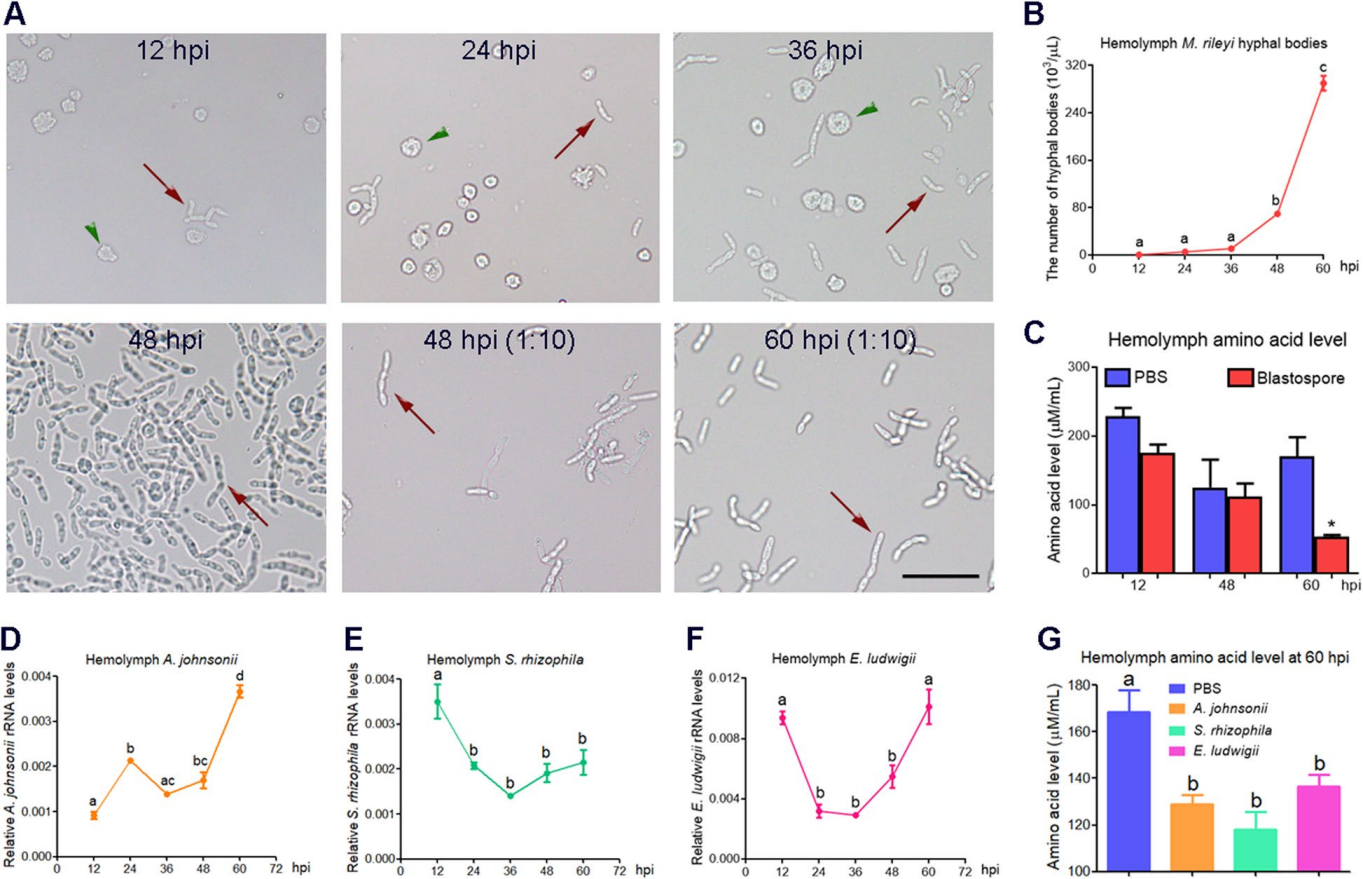
*M. rileyi* hyphal bodies or gut-derived bacteria with varied loads consume host amino acids from the hemolymph after infection. **A** Representative photographs indicate the hyphal bodies in the hemolymph of *H. armigera* larvae at 12, 24, 36, 48, and 60 hpi of *M. rileyi* blastospores. The hemolymph collected at 48 or 60 hpi was diluted (1:10) and photographed. The arrows indicate the hyphal bodies, whereas arrowheads represent the hemocytes. Scale bar = 50 μm. **B** The number of hyphal bodies in the hemolymph at 12, 24, 36, 48, and 60 hpi was determined. **C** The amino acid level in the hemolymph was measured at 12, 48, and 60 hpi of blastospores or PBS (control). **D-F** The varied load of *A. johnsonii* (**D**), *S. rhizophila* (**E**), or *E. ludwigii* (**F**) in the hemolymph from the larvae at 12, 24, 36, 48, and 60 hpi. The qPCR analysis was performed using specific primers of bacterial 16S rRNA gene and genomic DNA in the hemolymph as templates. **G** The amino acid level in the hemolymph was measured at 60 hpi of bacteria or PBS (control). The bacteria such as *A. johnsonii*, *S. rhizophila*, or *E. ludwigii* were injected individually. The data were analyzed by using Student’s *t* test (*p < 0.05) or one-way ANOVA followed by Tukey’s multiple comparison test. The different characters above the bars represent significant differences

By tracking the hyphal bodies in the hemolymph after blastospore injection, we found that the load of hyphal bodies remained low at 12, 24, and 36 hpi; however, the load of hyphal bodies increased significantly at 48 hpi and then sharply at 60 hpi (Fig. 10A, B). Meanwhile, the number of circulating hemocytes decreased at 48 h post-blastospore injection compared to the control group (Figure S4). The amino acid levels in the hemolymph remained unchanged between blastospore-injected and the control group at 12 and 48 hpi; however, they were significantly decreased at 60 hpi (Fig. 10C).

We also monitored the load of gut-derived bacteria following bacterial injection. The load of *A. johnsonii* in the hemolymph exhibited an increased trend overall with the prolongation of infection time (Fig. 10D). The load of hemolymph *S. rhizophila* decreased, whereas the load of hemolymph *E. ludwigii* first decreased and then increased (Fig. 10E, F). The amino acid levels in the hemolymph were significantly lower from the larvae injected with the gut-derived bacteria (*A. johnsonii*, *S. rhizophila*, or *E. ludwigii*) than that injected with PBS (Fig. 10G). These results indicated that either the hyphal bodies or the invasive gut bacteria required host amino acids in the hemolymph and there might have been competition between the two.

## Discussion

**Fig. 11:**
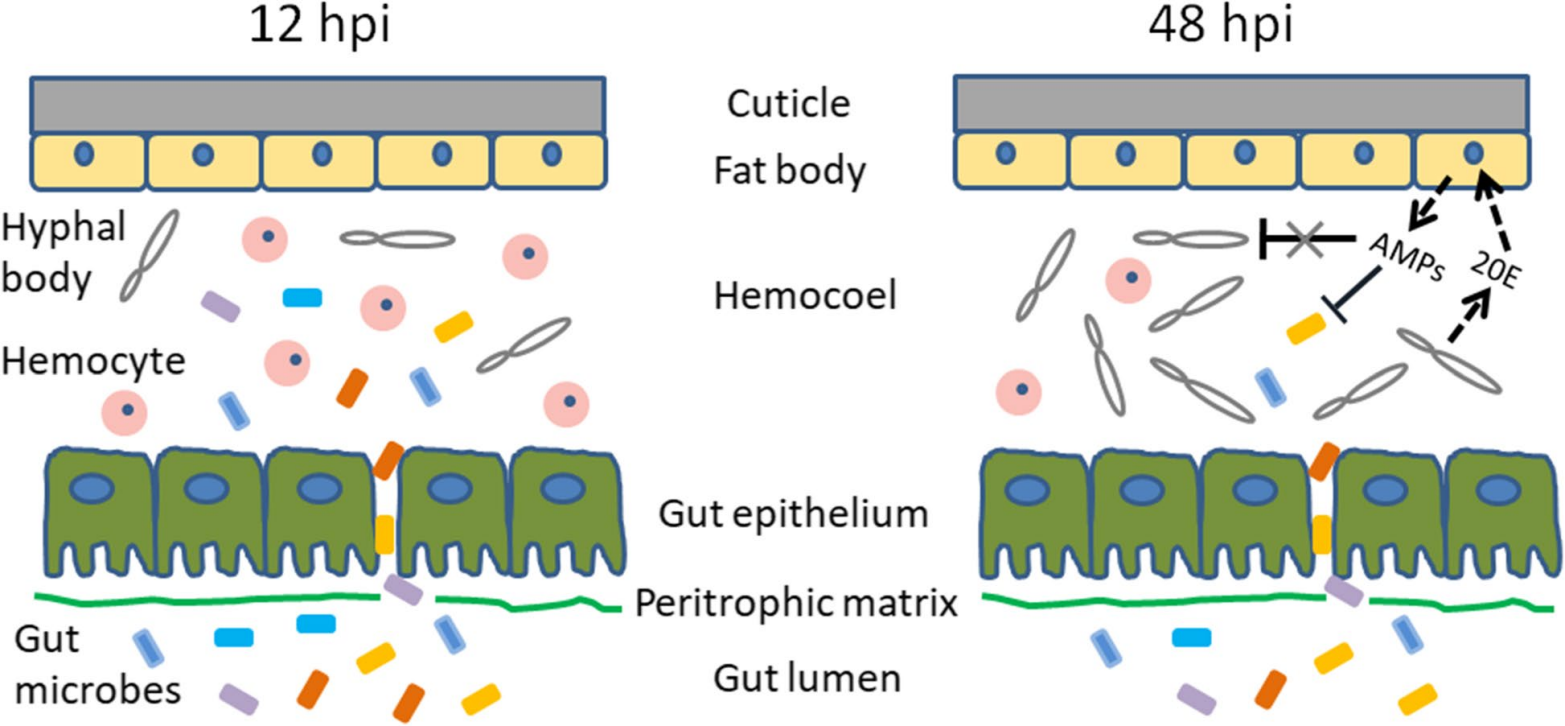
A proposed model for the translocation of gut bacteria into the hemocoel at 12 hpi of *M. rileyi* blastospores, followed by efficient elimination of these opportunistic bacteria at 48 hpi. The details in the text illustrate the involvement of *M. rileyi*-elevated 20E titers in inducing the expression of AMPs, which functions in the elimination of opportunistic bacteria. The efficient bacterial elimination therefore prevents opportunistic bacteria from competing with *M. rileyi* for the hemolymph amino acid nutrients required for the proliferation of hyphal bodies

An intriguing finding in the current study is that EPF utilizes host immunity to minimize the competition of opportunistic bacteria in the hemolymph. When EPF germ tubes penetrate the integument and enter the hemocoel, they are exposed to the strong immune reactions of the insect host [46, 47]. EPF usually employs two strategies to survive in the hemocoel: evading and suppressing the host immunity [6–10]. Here we have shown that *M. rileyi* infection causes gut bacteria to enter the hemocoel at 12 hpi, which are then quickly cleared at 48 hpi (Fig. 11). The invasive gut bacteria could compete with the hyphal bodies for amino acid nutrients. *M. rileyi*-elevated 20E contributes to the induction of AMPs, which exhibit potent inhibitory activity against invasive gut bacteria but not against hyphal bodies. Therefore, *M. rileyi* exploits host humoral antibacterial immunity to remove opportunistic bacteria, preventing them from competing for nutrients in the hemolymph. The exploitation of host immunity may represent a new strategy for EPF to interact with the host and complete its lifecycle.

Some studies have suggested that EPF infection leads to dysbiosis of gut microbiota, which contributes to the dysfunction of intestinal barrier and results in systemic inflammation [16, 19]. Song et al. [48] have shown that gut bacteria maintain PM structural integrity by regulating the expression of PM genes, such as *peritrophin* and *chitinase*. Therefore, it was likely that *M. rileyi* infection resulted in the dysbiosis of gut microbiota, which stimulated the expression of a gut chitinase *ChtEN03*, followed by impaired gut PM and leaking of bacteria from the gut to hemolymph. This was confirmed to some extent by the increased load of three characterized gut-derived bacteria in hemolymph at 12 h post-blastospore injection. Considering that bacterial and fungal chitinases also own the ability of increasing the permeability of PM [49, 50], we cannot rule out the direct role of gut bacteria or *M. rileyi* in breaking the PM, although the detailed mechanisms are still under exploration.

Interestingly, a significant decrease in hemolymph bacterial load and diversity at 48 h post-blastospore injection was observed, suggesting that invasive gut bacteria were largely eliminated. The load of three gut-derived bacteria in the hemolymph indeed decreased after fungal infection at 48 hpi. The involvement of gut bacteria in fungal pathogenesis may depend on insect taxa, its physiological state, fungal species, and dosages [19, 51–53]. While the gut microbiota promotes fungal pathogenesis, resulting in an accelerated death of mosquito after *B. bassiana* infection [19], no significant differences in survival is observed between the axenic and non-axenic *Bactrocera dorsalis* after *Metarhizium anisopliae* challenge [53]. Consistent with the latter, our data indicated that gut microbiota did not alter *M. rileyi* pathogenesis in *H. armigera* larvae. However, *M. rileyi* infection indeed increased the load of gut-derived bacteria in the hemolymph at 12 hpi. Since injection of gut-derived bacteria into hemocoel led to higher larval mortality, we believe that timely and efficient removal of invasive gut bacteria from hemolymph is the key to ensuring the healthy growth of larvae. A limitation of this study is that each species of bacteria (such as *A. johnsonii*) injected may account for a low proportion of invasive bacteria and cannot reflect the real situation. Cellular immune reactions are closely related to the number of circulating hemocytes [54]. Here, *M. rileyi* infection decreased the number of hemocytes at 48 hpi (Figure S4); therefore, it was unlikely that cellular immunity participated in the clearance of invasive gut bacteria in the hemolymph. As previously found in *Spodoptera frugiperda* [29], plasma antibacterial activity increased in *H. armigera* after *M. rileyi* infection, suggesting that this is a common phenomenon. Given that the plasma antibacterial activity was enhanced in the axenic larvae, we suggested that the enhanced plasma antibacterial activity may respond to *M. rileyi* rather than the invasive gut bacteria as previously proposed [29].

The increased expression of gloverin, cecropin 3, and lebocin may contribute to the enhanced plasma antibacterial activity in response to *M. rileyi* infection. The mutation of genes required for the activation of AMPs increases the sensitivity of *Drosophlia* to *B. bassiana* infection [55]. Cecropin A shows a high antifungal activity toward *B. bassiana* [56]. Although some AMPs are involved explicitly in defense against fungal infection [57], others are believed to have a spectral activity that can protect against the fungal and bacterial infections [58]. In addition to interacting with the membranes, AMPs also exert their antifungal activity by serving as cell wall inhibitors [57]. Considering that EPF sheds or masks the components of the cell wall as hyphal bodies [7, 8], this may be why cecropin 3 or lebocin could not suppress the proliferation of *M. rileyi* hyphal bodies.βGRPs usually bind to the fungal cell wall β-1,3-glucan and activate the Toll signaling pathway [3]. Since four βGRPs (βGRP2a, βGRP3, βGRP1, and βGRP2b) were upregulated in the plasma of *M. rileyi*-infected larvae, we hypothesized that the Toll pathway contributed to the AMP induction. This speculation was confirmed by the fact that gloverin, cecropin, and lebocin are involved in the Toll pathway [59, 60]. Some PRRs, such as CTLs, are involved in regulating AMPs [61, 62]. For example, we have previously shown that knockdown of CTL3 results in the downregulation of *cecropin*, *pre-gloverin*, and *lebocin* in *H. armigera* [28]. Therefore, we conclude that other PRRs, such as CTL3 identified here, may also contribute to the induction of AMPs. However, our previous study showed a lower affinity of βGRPs or CTL3 to *M. rileyi* hyphal bodies [27]. Given that ecdysone triggers the expression of many immune-related genes, such as PRRs and AMPs [42, 63], an increased 20E titer upon *M. rileyi* infection may contribute to the upregulation of AMPs, either directly or indirectly. The humoral antibacterial response of *H. armigera* upon fungal infection may be coordinated by the Toll pathway and ecdysone, as shown in the locust [47]. Unlike the decreased 20E levels upon *M. rileyi* conidial injection [41], we found that *M. rileyi* blastospore injection elevated the 20E titers. Conidia and blastospores represent different stages of fungal infection, thus possibly exerting different effects on host ecdysone levels. Considering that *M. rileyi* cells circulate as hyphal bodies (in vivo blastospores) in the hemolymph after penetration of the host integument [5], we believe that blastospore injection is closer to the natural infection.

*M. rileyi* utilizes nutrients like amino acids from the host’s hemolymph during infection, as did invasive gut bacteria, suggesting that the competition between EPF and gut bacteria exists. Injection of the gut bacteria into the hemocoel of *H. armigera* larvae decreased the number of hyphal bodies, further supporting the bacterial competition for hemolymph nutrition with EPF. Nutrition exploitation of host insects may be a fundamental feature for parasitic pathogens, as revealed previously [64, 65]. From an evolutionary point of view, eliminating the invasive gut bacteria can avoid competition for the nutrients and thus facilitate the growth and lifecycle of EPF. Fan et al. [25] revealed that *B. bassiana*, an EPF, has the ability to limit bacterial growth after host death and avoid bacterial competition on insect cadavers. Interestingly, our results indicate that *M. rileyi*, also an EPF, has evolved the capacity to outperform their bacterial competitors in absorbing host nutrients prior to host death. Unlike oosporein, a fungal secondary metabolite, employed by *B. bassiana* to restrict bacterial growth [25], host AMPs are induced and utilized by *M. rileyi* to eliminate bacterial challengers. Whether *M. rileyi* secretes secondary metabolites to limit invasive gut bacteria in the hemolymph remains unclear, at least not observed in in vitro coculture.

## Conclusions

Our studies have demonstrated that the gut bacteria translocate into the hemocoel following *M. rileyi* infection. *M. rileyi* triggers the expression of AMPs, thus eliminating competing opportunistic bacteria. It reduces bacterial competition by manipulating and exploiting the host’s humoral antibacterial immunity, thereby maximizing the absorption of nutrients from the host and facilitating the completion of the fungal lifecycle. We report for the first time the strategy and biological significance of fungi in eliminating opportunistic bacteria before insect death, which lays a theoretical foundation for the application of EPF in biological control.

## Supplementary Information

**Additional file 1:** **Figure S1. **Compromised peritrophic matrix (PM) integrity in *M. rileyi*-infected larvae. (A) The PM integrity was impaired in the larvae injected with blastospores. Paraffin section of the midgut from larvae at 12 hpi of blastospores or PBS was checked.The green arrow indicates intact PM, while the red arrow points to fragmented PM. Scale bar = 400 μm. (B) Comparison of differentially expressed genes (DEGs) in the midgut of blastospore-injected (MB) and PBS-injected (MP) larvae at 12 hpi. (C) RT-qPCR analysis of *ChtEN03 *expression in the midgut of larvae at 12, 24, and 48 hpi of blastospores or PBS. The statistical differences were analyzed using the Student’s *t* test (*p < 0.05 and **p < 0.01).

**Additional file 2:** **Figure S2.** Confirmation of the gut bacterial elimination by culturing gut homogenates on the LB agar plates (A) or conducting PCR assays using universal primers of bacterial 16S rRNA gene (B).

**Additional file 3:** **Figure S3.** The RT-qPCR analysis shows that the expression of *gloverin* (A), *cecropin 3* (B), and *lebocin *(C) was inhibited in whole at 24 and 48 hpi of gut-derived bacteria. *A. johnsonii*, *S. rhizophila*, and *E. ludwigii* were individually used for the challenge. The statistical differences were analyzed using the Student’s *t* test (*p < 0.05 and **p <0.01).

**Additional file 4:** **Figure S4.** *M. rileyi *infection decreases the number of hemocytes in *H. armigera* larvae. The number of circulating hemocytes was counted at 12, 24, and 48 hpi of blastospores or PBS (control). The statistical differences were analyzed using the Student’s *t* test (***p < 0.001).

**Additional file 5:** **Table S1.** The primers used in this study.

**Additional file 6:** **Table S2.** The proteins detected exclusively in the plasmacollected from PBS (PPB)-injected or blastospore (PBL)-injected groups at 48 hpi.

**Additional file 7:** **Table S3.** The proteins shared between the plasma collected from PBS (PPB)-injected and blastospore (PBL)-injected groups at 48 hpi.

**Additional file 8:** **Table S4.** The differentially expressed genes (DEGs) identified in the midgut of the larvae at 12 hpi of *M. rileyi* blastospores and PBS.

**Additional file 9:** **Table S5.** The gut and hemolymph operational taxonomic unit (OTU) taxonomy from the larvae at 48 hpi of *M. rileyi *blastospores or PBS.

## Data Availability

All data generated or analyzed during this study are included in this published article.
