# Supplementary figures and images for: An entomopathogenic fungus exploits its host humoral antibacterial immunity to minimize bacterial competition in the hemolymph
Source: Microbiome. 2023 May 20;11:116. doi: 10.1186/s40168-023-01538-6 (PMC10199594; doi:10.1186/s40168-023-01538-6)

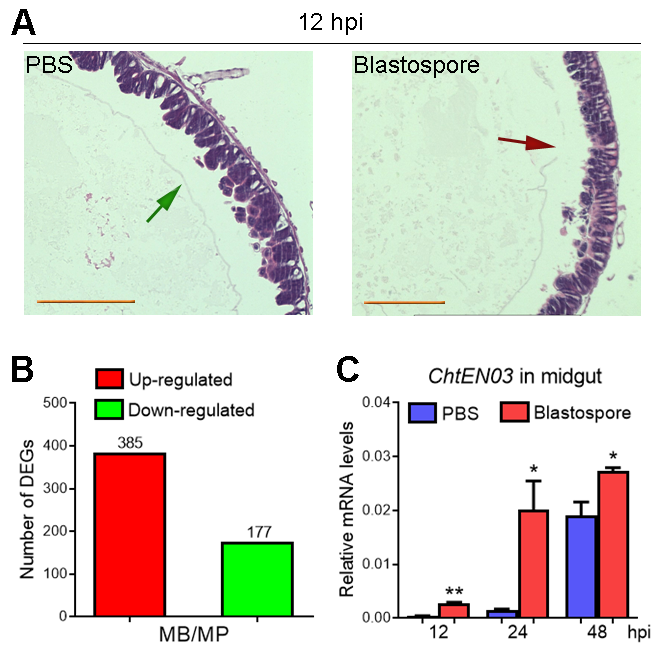

Supplement: Supplementary file 2 — Additional file 1: Figure S1. Compromised peritrophic matrix (PM) integrity in M. rileyi-infected larvae. (A) The PM integrity was impaired in the larvae injected with blastospores. Paraffin section of the midgut from larvae at 12 hpi of blastospores or PBS was checked.The green arrow indicates intact PM, while the red arrow points to fragmented PM. Scale bar = 400 μm. (B) Comparison of differentially expressed genes (DEGs) in the midgut of blastospore-injected (MB) and PBS-injected (MP) larvae at 12 hpi. (C) RT-qPCR analysis of ChtEN03 expression in the midgut of larvae at 12, 24, and 48 hpi of blastospores or PBS. The statistical differences were analyzed using the Student’s t test (*p < 0.05 and **p < 0.01). [file 40168_2023_1538_MOESM1_ESM.tif]

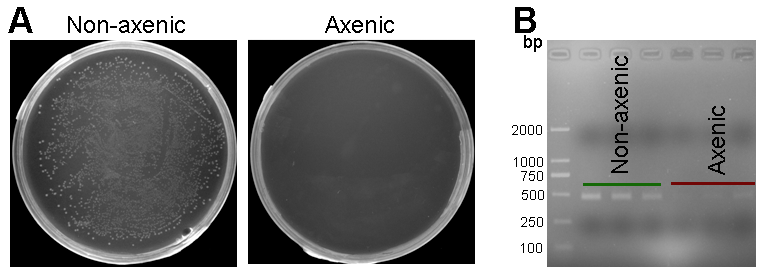

Supplement: Supplementary file 3 — Additional file 2: Figure S2. Confirmation of the gut bacterial elimination by culturing gut homogenates on the LB agar plates (A) or conducting PCR assays using universal primers of bacterial 16S rRNA gene (B). [file 40168_2023_1538_MOESM2_ESM.tif]

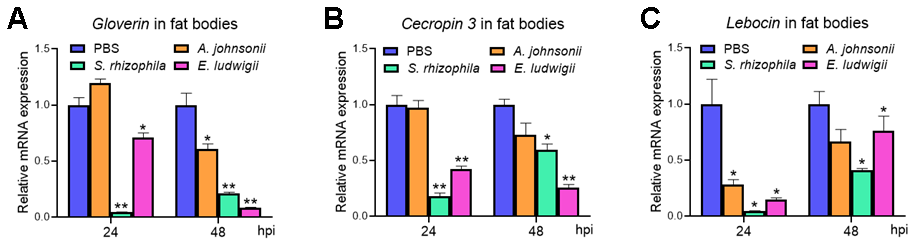

Supplement: Supplementary file 4 — Additional file 3: Figure S3. The RT-qPCR analysis shows that the expression of gloverin (A), cecropin 3 (B), and lebocin (C) was inhibited in whole at 24 and 48 hpi of gut-derived bacteria. A. johnsonii, S. rhizophila, and E. ludwigii were individually used for the challenge. The statistical differences were analyzed using the Student’s t test (*p < 0.05 and **p <0.01). [file 40168_2023_1538_MOESM3_ESM.tif]

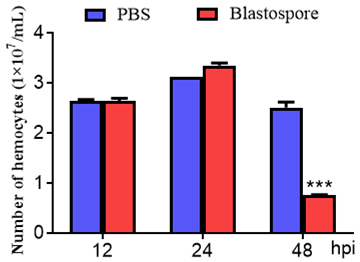

Supplement: Supplementary file 5 — Additional file 4: Figure S4. M. rileyi infection decreases the number of hemocytes in H. armigera larvae. The number of circulating hemocytes was counted at 12, 24, and 48 hpi of blastospores or PBS (control). The statistical differences were analyzed using the Student’s t test (***p < 0.001). [file 40168_2023_1538_MOESM4_ESM.tif]
